# Chemical Activation Boosted Interface Interaction between Poly(tetrafluoroethylene-co-hexafluoropropylene) Film and Silver Coating

**DOI:** 10.3390/polym16192730

**Published:** 2024-09-26

**Authors:** Hu Wang, Xiuqi Guo, Xuelei Li, Chenliang Gong, Yongqing Zhao

**Affiliations:** 1 Science and Technology on Vacuum Technology and Physics Laboratory, Lanzhou Institute of Physics, Lanzhou 730000, China; wanghu19841@163.com (H.W.); xueleili35910@sina.com (X.L.); 2 College of Chemistry and Chemical Engineering, Lanzhou University, Lanzhou 730000, China; guoxq20@lzu.edu.cn

**Keywords:** poly(tetrafluoroethylene-co-hexafluoropropylene), chemical activation, Ag coating, interface interaction

## Abstract

To enhance the interfacial adhesion between poly(tetrafluoroethylene-co-hexafluoropropylene) (FEP) film and functional coatings, such as silver (Ag) coating, among others, the surface activation of FEP film has to be performed. Among various activation strategies, chemical activation, such as using naphthalene sodium system, is one of the most efficient methods. However, the effect of chemical activation on the interface interaction between the activated FEP and functional coating is rarely investigated. Herein, the FEP film was activated by naphthalene sodium solution under different conditions, and then the Ag layer was coated onto its surface by vacuum Ag deposition. Based on experimental results and density function theory (DFT) calculation, it is indicated that oxygen-containing functional groups (such as C=O and C–OH groups), introduced onto the surface of FEP by the chemical activation, play a key role in boosting the interface interaction, which is due to the strong interaction between the oxygen-containing functional groups and Ag atoms. In addition, the concentration of naphthalene sodium solution, activation time, and winding speed of Ag- deposition can have a significant impact on the microstructures of Ag coating and the interfacial adhesion between the activated FEP and Ag coating. Under the conditions of high concentration (0.9 M), medium activation time (15 min), and high winding speed (0.8 m min^−1^), there is the best interface adhesion.

## 1. Introduction

Poly(tetrafluoroethylene-co-hexafluoropropylene) (FEP)/silver (Ag)-based second surface mirrors have many merits, such as low weight, good flexibility, excellent thermal radiation performance, and good stability in space environments. Therefore, it is one of the most important thermal control materials for various types of spacecraft, such as satellites, and has good application prospects in the development of long-life satellites in the future [1,2,3,4,5,6,7,8,9]. However, the surface-free energy of FEP is incredibly low, thus forming a non-viscous “low-energy surface”, which is attributed to the existence of a large number of fluorine (F) atoms in the molecular structure of FEP [10,11,12,13,14,15]. This makes weak binding force at the interface between FEP substrate and Ag coating, thus leading to loss of thermal control functions. This greatly limits the application of FEP in those specific fields. Evidently, in these application scenarios, it is necessary to treat the surface of FEP to improve surface energy, activate its surface, and thereby enhance its interface bonding with Ag coating.

At present, the most common methods for activating the surface of FEP include plasma treatment methods, chemical activation methods (such as using sodium naphthalene solution or magnesium with liquid ammonia solution), irradiation methods (light and electron irradiation), ion implantation methods, silicic acid modification methods, and mechanochemical treatment methods. These strategies have respective intrinsic advantages and disadvantages. For instance, plasma and irradiation treatments require expensive and complex equipment, while chemical methods and others display a drawback of potentially endangering the environment [16,17,18,19,20,21,22,23,24,25]. Among these methods, the sodium naphthalene solution system has attracted more attention and has been widely used, due to its simple process, low cost and satisfactory activation effect. Our previous work has discussed the effect of this method’s chemical activation conditions (such as concentration, solvent, and activation time) on the surface properties of the FEP film, and demonstrated that this strategy has superior activation performance than that of Ar plasma activation [26]. 

However, the effect of chemical activation on the interface interaction between the activated FEP and Ag coating is rarely investigated. Herein, the sodium naphthalene solution system was chosen to activate the surface of FEP thin films under different chemical activation conditions. In addition, Ag coating was deposited on the activated FEP films by using a pulsed DC reactive magnetron sputtering system. The effects of winding speed, sodium naphthalene solution concentration, and activation time on the surface microstructure of Ag coating and interfacial adhesion were studied. Furthermore, the interface enhancement mechanism of FEP/Ag was explored to provide a more comprehensive understanding of the effect of the chemical activation of FEP on vacuum Ag deposition. This work will help to optimize suitable activation conditions to achieve better interface enhancement effects and provide a basis for understanding the mechanism of enhancing the interfacial adhesion between FEP substrate and functional coatings.

## 2. Materials and Methods

### 2.1. Materials

1,3-dimethyl-2-imidazolinone (DMI), silver nitrate (AgNO_3_), ammonium hydroxide (NH_3_∙H_2_O), hydrazine hydrate (N_2_H_4_∙H_2_O), refined naphthalene, hydrochloric acid, absolute ethanol and acetone were purchased from Beijing Innochem Science & Technology Co., Ltd., Beijing, China. The silver target material with a purity of 99.99% was provided by the Beijing Nonferrous Metals Research Institute. All the reagents were used without further treatment.

### 2.2. Chemical Activation of FEP

The process of chemical activation for the FEP film (DuPont, Wilmington, NC, USA) was carried out according to our previous work [26]. Briefly, sodium naphthalene solution with different concentrations was prepared by dissolving metal sodium with different mass in a refined naphthalene–DMI solution. Then, FEP film was placed into sodium naphthalene solution with different concentrations (0.5, 0.7, or 0.9 M) and activated for different times (5, 15, or 30 min). After that, the FEP film was thoroughly washed with dilute hydrochloric acid, distilled water and absolute ethanol, and dried naturally. The activated FEP film samples were named as FEP-X-Y-Z. X, Y and Z represent the solvent, concentration of sodium naphthalene solution, and activation time, respectively.

### 2.3. Ag Coated onto FEP Film

The activated FEP film (thickness: 75 μm) was placed into the vacuum chamber of the magnetron sputtering coating equipment (1200RTR of Shengboer Photoelectric Technology Co., Ltd., Zhongshan, China), then vacuumized and started to be treated by ion beam. The vacuum chamber pressure, the argon flow rate, and ion source power were set as 0.15 Pa, 100 sccm, and 1200 W, respectively. In addition, the winding speed was chosen as 0.3, 0.5, or 0.8 m min^−1^. The FEP film activated with Ar plasma was assigned as FEP-P.

The chemical Ag deposition was carried out as follows. The pristine and chemically activated FEP (DMI-0.9 M-30 min) films were sonicated with acetone and ethanol for 30 min, respectively, and naturally dried in air. Then, the treated FEP films were immersed in a 0.01 M AgNO_3_ aqueous solution for 4 h to obtain an Ag seed layer onto their surfaces. After that, these FEP films were further placed into an ammoniacal silver solution (containing 5 mM [Ag(NH_3_)_2_]OH and 0.06 M hydrazine) to chemically deposit the Ag layer with different times (10, 20, 30, or 40 min) through Ag mirror reaction [27].

### 2.4. Characterization

Field-emission scanning electron microscopy (FE-SEM, Hitachi S4800, Hitachi, Tokyo, Japan) was used to investigate the surface and cross-section morphology of the FEP/Ag samples. X-ray diffraction (XRD) patterns were recorded through a Rigaku D/max-2400 diffractometer with Cu Kα radiation.

### 2.5. DFT Calculation

In order to further understand the interaction between activated FEP film and Ag coating, density functional theory (DFT) calculations were performed using the Dmol^3^ module [28] in the Materials Studio software package. Geometric optimizations were carried out by means of the Perdew–Burke–Ernzerhof [29] modification of the generalized gradient approximation [30] using the Grimme [31,32] correction with the doubled numerical basis set and polarization basis sets (including polarization *d*-function) [33]. The core electrons were dealt with by the DFT semi-core pseudo potentials. In addition, the global orbital cutoff and Fermi smearing were chosen as 3.2 Å and 0.005 Ha for the simulations, respectively. The convergence criteria were set as follows. The self-consistent field tolerance, maximum force tolerance, energy tolerance, and maximum displacement tolerance were 1.0 × 10^−5^ Ha per atom, 0.002 Ha Å^−1^, 1.0 × 10^−5^ Ha per atom, and 0.005 Å, respectively. Furthermore, the interaction energy (Δ*E*_int_) is defined as the difference between the total energy of the system and the energy sum of each component.

## 3. Results and Discussion

**Scheme 1:**
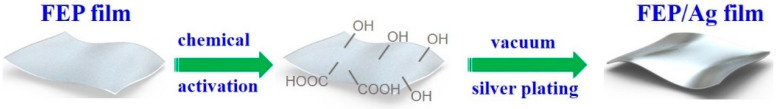
Schematic diagram of vacuum Ag deposition process on the surface of FEP activated by sodium naphthalene solution.

Our previous work has demonstrated that abundant C=O and C–OH groups can be easily introduced onto the surface of FEP film by the chemical activation of sodium naphthalene solution [26]. These oxygen-containing functional groups may have coordination or adsorption interactions with Ag atoms, which can provide a feasible and robust FEP substrate for vacuum Ag deposition (Scheme 1). The effects of the winding speed of the vacuum Ag deposition, sodium naphthalene solution concentration, and chemical activation time of FEP on the adhesion between FEP and Ag coating are discussed as follows.

### 3.1. The Effect of Winding Speed of Vacuum Ag Deposition

**Figure 1:**
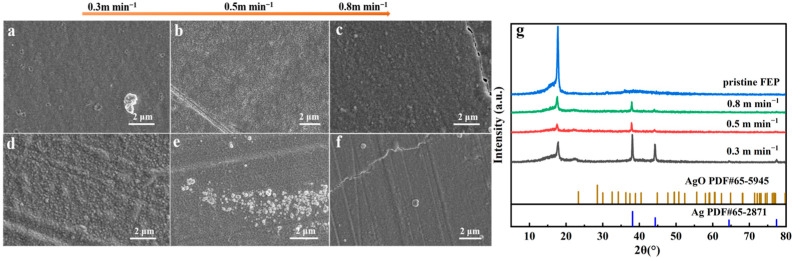
SEM images for surface morphology of vacuum Ag deposition samples at different winding speeds (from left to right, 0.3, 0.5, and 0.8 m min^−1^, respectively): (**a**–**c**) FEP-P, (**d**–**f**) FEP-DMI-0.9 M-30 min. XRD patterns of pristine FEP and vacuum Ag deposition samples (FEP-DMI-0.9 M-30 min) at different winding speeds (**g**).

Three winding speeds (0.3, 0.5, and 0.8 m min^−1^) were chosen for vacuum Ag deposition on different FEP films’ surfaces to investigate the effect of winding speed on the adhesion between FEP and Ag coating. As shown in Figure 1, attributed to the different winding speeds, whether on plasma-activated (FEP-P) or chemically activated (FEP-DMI-0.9 M-30 min) FEP films, Ag coating shows different surface morphology. At a high winding speed, the surface of Ag coating is relatively flat and cracks appear due to the thin Ag coating being unable to overcome internal stress (as shown in Figure 1c,f). At a low winding speed, the surface becomes increasingly rough. Meanwhile, it is observed that, under the same winding speed, the FEP activation method also has a significant impact on the morphology of the Ag coating. Especially at a low winding speed of 0.3 m min^−1^, the Ag coating on the FEP-P film is smoother and has some pores (Figure 1a), while the Ag coating on the FEP-DMI-0.9 M-30 min film is rougher with a large number of Ag nanoparticles (Figure 1d). This is likely attributed to the presence of different amounts of oxygen-containing functional groups on the FEP films’ surfaces activated by two different methods [26], which results in the different growth behavior of Ag. Furthermore, XRD was performed to obtain the structure information of these samples (Figure 1g). The diffraction peaks at 17.8° can be assigned to the (100) reflection of FEP [34]. The diffraction peaks also appear at 38.1° and 44.3°, owing to the (111) and (200) characteristic diffraction peaks of Ag, respectively. It is noted that the diffraction peaks of silver oxides are hardly observed. 

**Figure 2:**
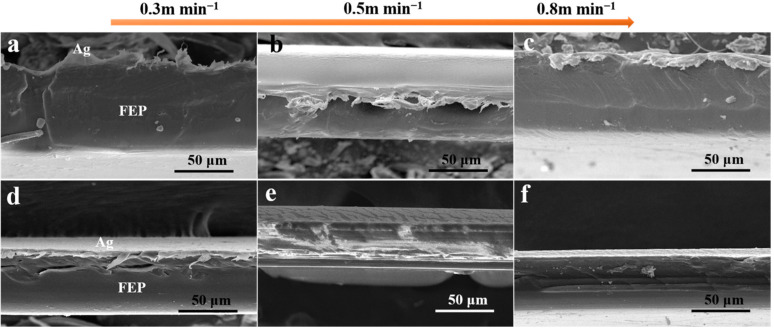
SEM images for fracture surface of vacuum Ag-deposition samples at different winding speeds (from left to right, 0.3, 0.5, and 0.8 m min^−1^, respectively): (**a**–**c**) FEP-P, (**d**–**f**) FEP-DMI-0.9 M-30 min.

In further experiments, the influence of activation methods on the interface structure between the activated FEP films and Ag coating was investigated, and the samples were cut by scissors to obtain the fracture surface. As shown in Figure 2a–c, under different winding speeds, the Ag coating is severely detached from the FEP-P substrate. In contrast, it can be seen from Figure 2d–f that the Ag coating is tightly bound to the chemically activated sample (FEP-DMI-0.9 M-30 min), especially at high winding speeds. This may be attributed to the presence of more oxygen-containing functional groups on the chemically activated sample surface, which facilitates the formation of interactions with the Ag coating, thus enhancing the bonding force between the two. Evidently, the chemical activation method is more efficient for boosting the interface bonding between the Ag coating and FEP film.

### 3.2. The Effect of Sodium Naphthalene Solution Concentration

**Figure 3:**
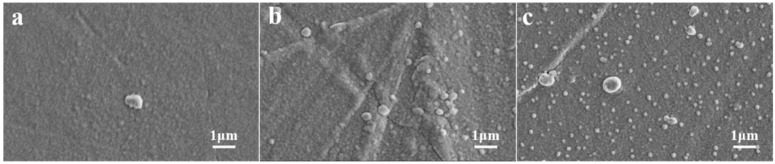
SEM images for surface morphology of vacuum Ag deposition samples at a winding speed of 0.3 m min^−1^, where FEP was activated by sodium naphthalene solution with different concentrations: (**a**) FEP-DMI-0.5M-15min, (**b**) FEP-DMI-0.7 M-15 min, (**c**) FEP-DMI-0.9 M-15 min.

As shown in Figure 3, the concentration of sodium naphthalene solution also has a significant impact on the surface morphology of vacuum Ag deposition samples. When the concentration is increased, the activation degree of the FEP surface is intensified, and thus more oxygen-containing groups are introduced onto the surface [26]. This may affect the deposition and crystallization processes of Ag atoms on the FEP surface, which leads to the gradual generation of a large number of Ag nanoparticles on the FEP surface. 

**Figure 4:**
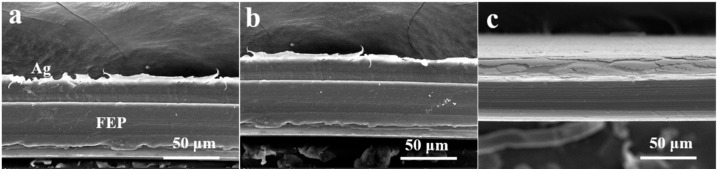
SEM images for fracture surface of vacuum Ag deposition samples at a winding speed of 0.3 m min^−1^, where FEP was activated by sodium naphthalene solution with different concentrations: (**a**) FEP-DMI-0.5 M-15 min, (**b**) FEP-DMI-0.7 M-15 min, (**c**) FEP-DMI-0.9 M-15 min.

Figure 4 displays the fracture interface structure between the Ag coating and activated FEP films with different concentrations of sodium naphthalene solution. It can be seen that the Ag coating is seriously detached from the FEP substrate activated with a low concentration of sodium naphthalene solution (Figure 4a,b), while the cross-section of the FEP-DMI-0.9 M-15 min sample shows better adhesion between the Ag coating and the FEP substrate (Figure 4c). This indicates that increasing the concentration of sodium naphthalene solution is beneficial to improving the interfacial adhesion between the Ag coating and activated FEP substrate, thus making the interface structure more stable. When the FEP film was activated by high concentration sodium naphthalene solution, more oxygen-containing functional groups could be introduced onto its surface, thus probably forming adsorption interactions between the FEP film and Ag atoms. 

### 3.3. The Effect of Chemical Activation Time

**Figure 5:**
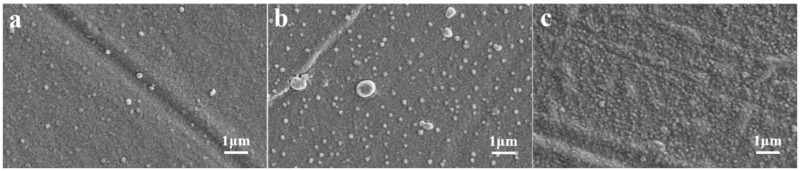
SEM images for surface morphology of vacuum Ag deposition samples at a winding speed of 0.3 m min^−1^, where FEP was activated by sodium naphthalene solution for different times: (**a**) FEP-DMI-0.9 M-5 min, (**b**) FEP-DMI-0.9 M-15 min, (**c**) FEP-DMI-0.9 M-30 min.

In addition, the chemical activation time can also affect the surface morphology of vacuum Ag deposition samples on the activated FEP substrate. As shown in Figure 5, with the increase in activation time, the surface of the Ag coating becomes rougher, and more Ag nanoparticles gradually appear. This phenomenon may also be due to the intensified activation on the FEP substrate with increasing activation time; thereby, more oxygen-containing groups change the deposition and crystallization mode of Ag atoms on the FEP surface.

**Figure 6:**
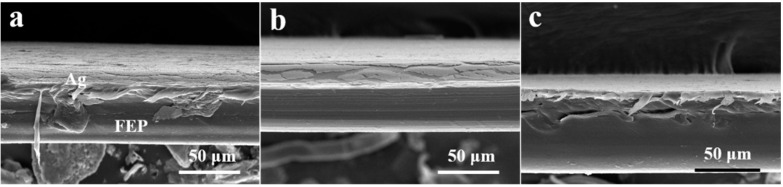
SEM images for fracture surface of vacuum Ag deposition samples at a winding speed of 0.3m min^−1^, where FEP was activated by sodium naphthalene solution for different times: (**a**) FEP-DMI-0.9 M-5 min, (**b**) FEP-DMI-0.9 M-15 min, (**c**) FEP-DMI-0.9 M-30 min.

Figure 6 clearly displays the detachment of Ag coating from the FEP substrate is more severe at the cross-sections of FEP-DMI-0.9 M-5 min (Figure 6a) and FEP-DMI-0.9 M-30 min (Figure 6c) samples. Although the Ag coating on the cross-section of the FEP-DMI-0.9 M-15 min (Figure 6b) sample is broken, it is not detached from the FEP substrate, indicating that, under this activation condition, the interface bonding force between the Ag coating and the activated FEP substrate is stronger and the interface structure is more stable. This also suggests that selecting an appropriate activation time can improve the interfacial adhesion between the Ag coating and FEP substrate. The shorter activation time generates fewer oxygen-containing functional groups on the activated FEP surface and weaker interactions with the Ag coating; When the activation time is prolonged, the surface of the FEP is too rough, which weakens the bonding force between the Ag coating and the FEP substrate, leading to unstable interface structure.

### 3.4. Mechanism of Enhanced Interface Interaction by Chemical Activation

**Figure 7:**
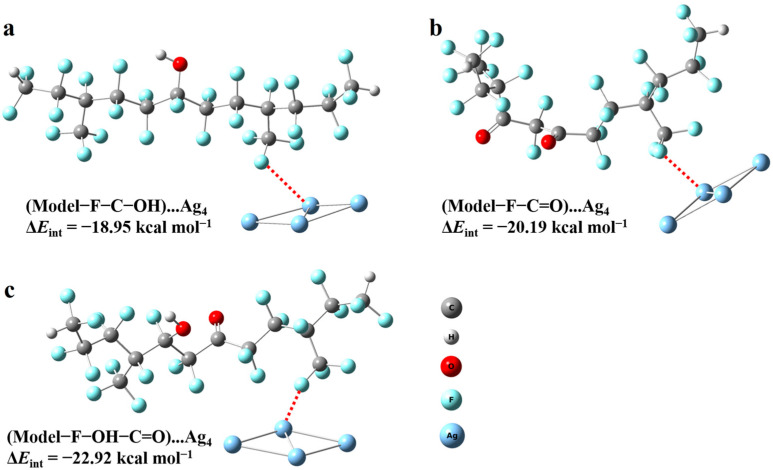
Schematic illustration for DFT calculations of the interaction energy between silver atoms and chemically activated FEP with different oxygen-containing functional groups: (**a**) FEP with –OH groups, (**b**) FEP with –C=O groups, (**c**) FEP with –OH and –C=O groups.

Due to the fact that a large number of C=O and C–OH groups can be introduced onto the surface of FEP by chemical activation [26], interactions may be generated between the oxygen-containing functional groups and Ag atoms, thus improving the adhesion between FEP and Ag coating. Bucio et al. have demonstrated that there is an interaction between the −OH groups and Ag nanoparticles [35]. In order to understand their interactions, DFT calculations were performed. As shown in Figure 7, three models were constructed for investigating the interaction between Ag clusters composed of 4 Ag atoms and FEP with C–OH and/or C=O groups. The simulation calculation between Ag_4_ and the chemically activated FEP shows that Ag_4_ is adsorbed on the chemically activated FEP, and the adsorption interaction between Ag_4_ and the chemically activated FEP is schematically represented using the red dotted line. Furthermore, the interaction energies (Δ*E*_int_) of three models are −18.95, −20.19, and −22.92 kcal mol^−1^, respectively. This indicates that the presence of different oxygen-containing functional groups will generate different adsorption interactions between the Ag clusters and activated FEP. Evidently, the adsorption interaction is strongest when −OH and C=O groups coexist on the activated FEP [36].

**Figure 8:**
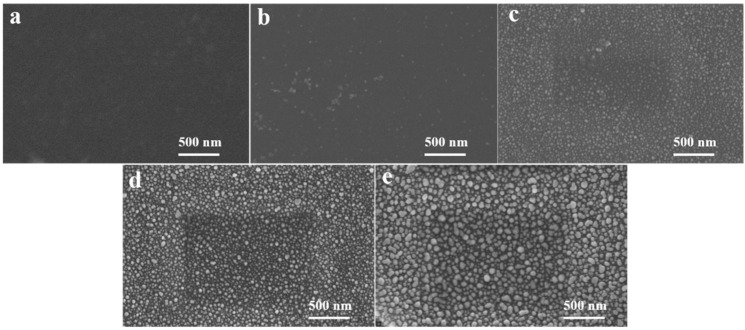
SEM images for chemical Ag deposition onto the pristine FEP and FEP-DMI-0.9 M-30 min: (**a**) pristine FEP for 30 min; FEP-DMI-0.9 M-30 min for (**b**) 10 min, (**c**) 20 min, (**d**) 30 min, (**e**) 40 min.

To further verify the good affinity between the chemically activated FEP surface and Ag, we conducted chemical Ag deposition on the surface of pristine FEP and FEP-DMI-0.9 M-30 min. As shown in Figure 8a, after 30 min of chemical Ag deposition on the pristine FEP surface, there are almost no Ag nanoparticles observed on its surface. Meanwhile, under the same chemical Ag deposition conditions, a large number of Ag nanoparticles (approximately 50 nm in size) uniformly appear on the surface of FEP-DMI-0.9 M-30 min (Figure 8d). In addition, with the increase in chemical Ag deposition time from 10 to 40 min, the growth amount of Ag nanoparticles on the FEP-DMI-0.9 M-30 min gradually increases and Ag nanoparticles maintain good dispersion and particle size uniformity (Figure 8b–e). Evidently, the results of the chemical Ag deposition experiments can further confirm that chemically activated FEP has a better affinity between its surface and Ag, thus enhancing the interfacial bonding between the two.

## 4. Conclusions

In summary, the chemical activation conditions (such as sodium naphthalene solution concentration and activation time) and winding speed of vacuum Ag deposition can have a significant impact on the Ag deposition process and interface interaction between the activated FEP substrate and the Ag coating. Based on DFT calculations and experimental results, it is confirmed that the chemically activated FEP substrate has better adhesion with the Ag coating than that of the Ar plasma-treated FEP substrate. That is, chemical activation can effectively enhance the adhesion between the FEP substrate and the Ag coating. Under the conditions of high concentration of sodium naphthalene solution, appropriate chemical activation time, and high winding speed of vacuum Ag deposition, the best interfacial adhesion can be obtained. Furthermore, the chemical activation method has the advantages of low cost, simplicity, and short treatment time. Therefore, the chemical activation method is a potential optional strategy for surface activation of FEP used as a robust substrate for vacuum Ag deposition. 

## Data Availability

The data presented in this work are available on request from the corresponding authors.
